# The structural characterization and UV-protective properties of an exopolysaccharide from a *Paenibacillus* isolate

**DOI:** 10.3389/fphar.2024.1434136

**Published:** 2024-08-09

**Authors:** Xiaodong Xu, Zhao Ding, Chunlin Pu, Changchang Kong, Shijunyin Chen, Weiling Lu, Jianfa Zhang

**Affiliations:** ^1^ Center for Molecular Metabolism, Nanjing University of Science & Technology, Nanjing, China; ^2^ Key Laboratory of Metabolic Engineering and Biosynthesis Technology, Ministry of Industry and Information Technology, Nanjing, China

**Keywords:** exopolysaccharide, Paenibacillus, UVB radiation, natural sunscreen, antioxidant

## Abstract

**Introduction:**

Overexposure to ultraviolet (UV) light is known to cause damage to the skin, leading to sunburn and photo-aging. Chemical sunscreen products may give rise to health risks including phototoxicity, photosensitivity, and photosensitivity. Natural polysaccharides have attracted considerable interests due to diverse biological activities.

**Methods:**

A novel polysaccharide isolated was purified and structurally characterized using chemical methods followed by HPLC, GLC-MS, as well as 1D and 2D NMR spectroscopy. The photoprotective effect of the EPS on UVB-induced damage was assessed *in vitro* using cultured keratinocytes and *in vivo* using C57BL/6 mouse models.

**Results:**

The average molecular weight of the EPS was 5.48 × 10^6^ Da, composed of glucose, mannose and galactose residues at a ratio of 2:2:1. The repeating units of the EPS were →3)-β-D-Glc*p* (1→3) [β-D-Gal*p* (1→2)-α-D-Glc*p* (1→2)]-α-D-Man*p* (1→3)-α-D-Man*p* (1→. In cultured keratinocytes, the EPS reduced cytotoxicity and excessive ROS production induced by UVB irradiation. The EPS also exhibits an inhibitory effect on oxidative stress, inflammation, and collagen degradation found in the photodamage in mice. ^1^H NMR-based metabolomics analysis for skin suggested that the EPS partly reversed the shifts of metabolic profiles of the skin in UVB-exposed mice.

**Conclusion:**

The EPS exhibits skin photoprotective effects through regulating oxidative stress both *in vivo* and *in vitro*. Our findings highlight that the EPS is a potential candidate in sunscreen formulations for an efficient solution to UVB radiation.

## 1 Introduction

Ultraviolet (UV) radiation from the sun is an essential part of life and has many beneficial effects on human health, such as vitamin D synthesis (Holick, 2020). However, overexposure to UV radiation can cause damage to the skin, eyes and immune system, leading to skin cancer, premature aging and other health problems (Amaro-Ortiz et al., 2014). Particularly the shorter wavelength Ultraviolet B (UVB) can induce various detrimental effects on biological systems, including DNA damage, oxidative stress and inflammation, which are associated with skin cancer and premature aging (Jacovides et al., 2009). According to the World Health Organization, more than 1.5 million cases of skin cancer were diagnosed and over 120,000 deaths associated with skin cancer were reported globally in 2020 (World Health Organization, 2023).

UV photons cause skin damage through two mechanisms: direct absorption and photosensitization. In the direct absorption mechanism, the cellular chromophores absorb UV radiation and transform the absorbed energy into a biochemical signal, which induces apoptosis of irradiated keratinocytes. On the other hand, in the photosensitization mechanism, sensitizers absorb UV light, leading to the formation of Reactive Oxygen Species (ROS) (Saewan and Jimtaisong, 2015). Considering the harmful effects, it is essential to employ an appropriate strategy to mitigate UV-induced damage. Recent studies have uncovered polysaccharides derived from natural sources with potential for protecting against UV damage (Rastogi et al., 2015; Hu et al., 2019; Michalak, 2022). Additionally, these polysaccharides have been shown to prevent skin aging and improve skin hydration (Chen et al., 2016; Li et al., 2022a). Several studies have indicated that the antioxidant and anti-inflammatory properties of polysaccharides may alleviate UVB-induced skin damage (Lu et al., 2023). Bacterial exopolysaccharides (EPSs) offer advantages over plant polysaccharides, as they can be produced in larger quantities at a lower cost and extracted more simply. Compared to traditional chemical sunscreens, EPS exhibit non-toxic, biodegradable and renewable, making them more environmentally friendly (Singh et al., 2023). These distinct properties make EPS an attractive option for use in sunscreens. EPS exhibit a wide range of structural diversity, including differences in monosaccharide composition, branching patterns, and glycosidic linkages. The biological activities of EPS are greatly influenced by their structure (Ali et al., 2024).

Bacteria belonging to the genus *Paenibacillus* have been isolated from a variety of environments, with the majority being found in soil (Grady et al., 2016). Different EPS from *Paenibacillus* strains are usually composed of glucose, mannose, galactose and glucuronic acid in various mole ratios (Liang and Wang, 2015). The EPS from *Paenibacillus polymyxa* SQR-21 is comprised of mannose, galactose and glucose (Raza et al., 2011). The EPS produced by *P. polymyxa* PYQ1 is comprised of mannose, ribose, glucuronic acid, glucose, galactose and xylose. These dissimilarities reflect the species-specific structure and biotechnological potential of EPS. The *Paenibacillus* strains produce a wide variety of different EPS with diverse physiological and biotechnological functions, including antioxidant, antitumor, skin hydration improvement, and wastewater bioremediation functions as reported in previous studies (Liang and Wang, 2015). For example, those EPS from *P. polymyxa* SQR-21 and *P. polymyxa* EJS-3 have superoxide scavenging activity and inhibit lipid peroxide. The EPS from *P. polymyxa* PYQ1 has the protective benefits against UVC induced cytotoxicity in keratinocyte cells through scavenging excessive ROS, mitigating the decrease of mitochondrial membrane potential, improving catalase activity and maintaining membrane integrity (Wang et al., 2020).

The growing interest in natural and organic sunscreens is also a positive trend that can benefit both human health and the environment. By utilizing sunscreen correctly and adopting other protective measures, we can minimize the risks of UV damage and enjoy the benefits of the sun safely (Young et al., 2017; Narla and Lim, 2020). In this study, we isolated an EPS-producing bacterial strain from a forest soil sample. The 16s rDNA sequence of this strain was analyzed and compared to other bacteria. The structural characteristics of the EPS was determined using various techniques such as high-performance liquid chromatography (HPLC), gas chromatography-mass spectrometry (GC–MS) and nuclear magnetic resonance (NMR). We investigated the capacity of the EPS to protect against UVB-induced damage in both HaCaT cells and mouse models. Using these assays, we aimed to evaluate the protective potential of EPS in UVB-induced skin damage and examined the underlying mechanisms.

## 2 Materials and methods

### 2.1 Materials and reagents

Forest soil samples was collected from Nanjing City, Jiangsu Province, China (locality: 32° 04′N,118° 86′E). D-glucose (D-Glc), D-mannose (D-Man), D-galactose (D-Gal) and D_2_O (#7789-20-0) were purchased from Aladdin Co., Ltd (Shanghai, China). Genomic DNA purification kit (#K2303) was purchased from Nanjing Karroten Biotechnology Co., Ltd (Nanjing, China). Lowry method protein concentration determination kit (#C504031-1001) was purchased from Sangon Biotech Co., Ltd (Shanghai, China). Dextran standards (ranging from 21 to 2,990 kDa, #DXT21K, #DXT97K, #DXT300K, #DXT820K, #DXT2990K) were purchased from American Polymer Standards Corporation. RNA reverse transcriptase (#G490) was purchased from ABM (Canada). SYBR Green real time PCR mix (#QPK201) was purchased from Toyobo company ltd. (Osaka, Japan). Superoxide dismutase (SOD) (#A001-3-2), malondialdehyde (MDA) (#A003-1-2), malondialdehydecatalase (CAT) (#A007-2-1), glutathione (GSH) (#A006-2-1) and glutathione peroxidase (GSH-Px) (#A005-1-2) test kits were purchased from Nanjing Jiancheng Bioengineering Institute (Nanjing, China). Cell Counting Kit (CCK-8) were purchased from Beyotime Biotechnology Co., Ltd. (Shanghai, China).

### 2.2 Bacteria and growth conditions

Bacterial strain sp.1538 was grown in 50 mL of HBN medium (MnSO_4_·H_2_O 0.003 g/L, FeSO_4_.7H_2_O 0.0125 g/L, ZnCl_2_ 0.0075 g/L, CaCl_2_ 0.07 g/L, MgSO_4_·7H_2_O 0.2 g/L, Na_2_HPO_4_ 1.5 g/L, KNO_3_ 0.2 g/L, sucrose 30 g/L), pH 8.0. Strain cultivation was maintained at 30°C for 2 days. Genomic DNA was extracted from bacterial strain sp.1538 using a genomic DNA purification kit, followed by amplification and sequencing of the 16S rRNA gene by GENEWIZ Inc (Suzhou, China). The obtained sequence was compared to sequences in the GenBank database using the Basic Local Alignment Search Tool (BLAST) to identify the closest phylogenetic match (Xiao et al., 2020). The strain was deposited in the China Center for Type Culture Collection (CCFMC) with the storage number CCFMC NO: M20221644. The OD_600_ of ferment broth was measured in real-time by a Bacterial Growth Curves Meter (Karroten Scientific, Nanjing, China).

### 2.3 Extraction and purification of the EPS

The EPS isolation and purification were carried out according to the procedure described (Kavitake et al., 2016) with slight modification. The EPS was extracted by collecting the bacterial culture through centrifugation, and the resulting supernatant was retained. Then the EPS was precipitated with cold ethanol (three times the volume) and left overnight. The precipitate was collected and dissolved in distilled water. The crude EPS was purified using Sevag reagent (trichloromethane: butanol = 4:1, v/v) to remove proteins, followed by precipitation with cold ethanol (three times its volume). The precipitate was then dried in a vacuum oven for 24 h at 40°C.

### 2.4 Chemical composition analysis

The protein content was quantified using the Folin-Lowry method, with bovine serum albumin as the standard (Sapan et al., 1999). Total sugar content was determined using the phenol-sulfuric acid method, with glucose as the reference (Yue et al., 2022).

### 2.5 Molar mass determination

The average molecular weight (M_W_) of the EPS was determined as described previously (Chen S. et al., 2022). In short, a sample (1 mg/mL) was applied to a TSK gel Super Multipore PW-H column (6.0 mm × 150 mm, Tosoh Corp., Japan). The mobile phase, comprising sodium phosphate buffer (15 mM, pH 5.0) with 0.03% sodium azide (NaN_3_) at a flow rate of 0.6 mL/min, was used for elution, and the eluate was analyzed using a refractive index detector. Dextran standards (21, 97, 300, 820, and 2,990 kDa) were prepared to establish the calibration curve.

### 2.6 FT-IR and UV-vis spectroscopic analysis

Fourier Transform Infrared Spectroscopy (FT-IR) was performed on the purified EPS sample (Li et al., 2023). The sample was analyzed using a Fourier transfer infrared spectroscope (Nicolet iS20, Thermo Scientific). UV-vis absorption spectrum of the EPS was performed using a microplate reader (PowerWave XS, BioTek, German).

### 2.7 Monosaccharide composition

The monosaccharide composition of the EPS was analyzed using reverse-phase HPLC following 1-phenyl-3-methyl-5-pyrazolone (PMP) derivatization procedures (Singh et al., 2019; Zhang et al., 2021). After sealing under N_2_, 1 mL of 2 M trifluoroacetic acid (TFA) was added, and the solution was then hydrolyzed at 100°C for 6 h and recovered by vacuum desiccation. The PMP derivatives were filtered through a 0.22 μm microporous membrane and analyzed using a Waters HPLC system equipped with a Zorbax SB-Aq column. The mobile phases A and B (82.3/17.7, v/v) consisted of 0.03 M KH_2_PO_4_ (pH 5.6) and acetonitrile, respectively, with a flow rate of 0.9 mL/min, and the absorbance was monitored at 248 nm.

### 2.8 Methylation analysis

Methylation analysis was performed to identify the glycosidic linkage of the EPS, following previously described methods with minor modifications (Chen S. et al., 2022; Hibberd et al., 2024). The EPS sample was reacted with saturated NaOH and iodomethane in DMSO. Residual NaOH was removed by extraction with chloroform and water. The organic layer was dried under a stream of nitrogen. Methylation was repeated once and hydrolyzed with 4 M TFA for 2 h at 100°C. Partially methylated monosaccharides were reduced using NaBH_4_ and acetylated with acetic anhydride and pyridine. The partially methylated alditol acetates (PMAAs) were separated and identified by a GC-MS system (Thermo Scientific Trace1300 ISQ, United States) equipped with a TG-200MS capillary column (0.25 mm × 0.25 mm × 300 mm). The parameters are set as follows: 50°C for 1 min, followed by an increase of 40°C/min up to 130°C, and a further increase of 3°C/min up to 230°C with a 2 min hold. Helium was used as the carrier gas. The scanning mode was SCAN, and the range (m/z) was 50–350.

### 2.9 NMR analysis of the EPS

For NMR analysis, the sample was hydrolyzed in 0.5 M TFA at 100°C for 1 h (Gaikwad et al., 2023). Subsequently, the TFA was removed, and the solution was dialyzed (Mw cutoff: 500 Da) and then lyophilized. The 20 mg sample underwent deuterium exchange through two freeze-drying cycles from D_2_O. Finally, the sample was examined as a solution in D_2_O (80 mg/mL), with trimethylsilyl propionate (TMSP) utilized as the internal standard. The ^1^H, ^13^C, HSQC, HMBC, COSY, TOCSY and NOESY spectra of the EPS were recorded by 500 MHz spectrometer manufactured by Bruker (Karlsruhe, Germany). In addition, ^1^H NMR spectra of a mildly partially acid-hydrolyzed EPS sample after reducing the hydrolysis time from 1 h to 0.5 h was also recorded. The ^1^H NMR spectra was recorded with a standard pulse program, relaxation delay of 1 s, 32 scans and an acquisition time of 3.28 s. The ^13^C NMR data were obtained with a relaxation delay of 2 s, 5,120 scans and an acquisition time of 1.1 s. The data was processed using the Bruker Topspin 4.0 software package (Kokoulin et al., 2022). The mixing time of 120 ms and 300 ms was used in the TOCSY and NOESY experiments, respectively. The acquired spectra were Fourier transformed, phased, and baseline corrected. The chemical shift assignments were performed based on standard reference compounds and by comparison with previous literature.

### 2.10 Cell culture and determination of cell viability

HaCaT cells (human keratinocyte cell line) and NIH3T3 cells (murine fibroblast cell line) were kindly provided by Stem Cell Bank, Chinese Academy of Sciences, cultured in DMEM supplemented with 10% FBS and 1% penicillin/streptomycin. Cells were kept at 37°C with 5% CO_2_ in a FORMA SERIES 3 WATER JACKETED CO_2_ Incubator (Thermo Scientific, United States).

The cells were seeded at a density of 8 × 10^3^ cells per well in a 96-well plate and incubated for 24 h NIH3T3 and HaCaT cells were treated with or without the EPS at various concentrations (0–200 μg/mL). After 24 h, the cell viability was evaluated. HaCaT cells and NIH3T3 cells were placed below a UVB light source (SGBE-118) at a distance of 20 cm for 100, 200 and 300 s to 20, 40, and 60 mJ/cm^2^ of the irradiation dose. Cells were evaluated by CCK-8 assay at 24 h after UVB irradiation. To assess the protective effect of the EPS against UVB-induced damage to HaCaT cells and NIH3T3 cells, the cells were pre-treated with various concentrations of the EPS (0–200 μg/mL) and incubated for 1 h before exposing them to UVB (20 mJ/cm^2^). After 24 h incubation, cell viability was also evaluated. The CCK-8 assay was performed following the method provided by the commercial kit.

### 2.11 FITC conjugates and fluorescence analysis

The EPS was labeled with Fluorescein Isothiocyanate (FITC) as previously described (Zheng et al., 2020). Briefly, to prepare the FITC- EPS conjugate, 10 mg of EPS was dissolved in 100 mL of DMSO with 3 drops of pyridine. FITC (20 mg) and dibutyltin dilaurate (20 mg) were added, mixed and reacted at 95°C for 2 h. After removal of excess reagents by ethanol washing, the EPS-FITC conjugate was dialyzed with distilled water (M_W_ cutoff: 10 kDa) and then freeze-dried for further use. NIH3T3 and HaCaT cells were treated with FITC-labeled EPS for 1 h. The fluorescence images were scanned using a fluorescence microscope (Nikon, Tokyo, Japan).

### 2.12 Determination of intracellular ROS generation and antioxidant activities

HaCaT cells were cultured in 6-well tissue culture plates (2 × 10^5^ cells per well) and divided into four groups: (1) normal group (Control), neither UV exposure nor any treatment; (2) UVB-irradiated group (UVB), which received 20 mJ/cm^2^ UVB exposure alone; (3) low dose of EPS group (LP), which treated 50 μg/mL EPS prior to UVB exposure; (4) high dose of EPS group (HP), which treated 100 μg/mL EPS prior to UVB exposure. Cells were exposed to UVB (20 mJ/cm^2^) for 1 h, 2′7′-dichlorofluorescein diacetate (DCFH-DA) fluorescent probe was used to assess intracellular ROS generation in HaCaT cells. The cells were then incubated in fresh culture media for 24 h. Subsequently, cells were stained with DCFH-DA probe under a humidified atmosphere (5% CO_2_) at 37°C for 30 min. The cells were observed under a fluorescence microscope and the DCFH-DA fluorescence intensity was analyzed with a NovoCyte flow cytometer (ACEA Bioscience Inc. San Francisco, CA, United States). SOD, CAT, GSH-Px, GSH, and MDA levels were detected using commercial assay kits (Nanjing Jiancheng Bioengineering Institute, China).

### 2.13 Animals and UVB irradiation

Mice were purchased from Jiangsu Jichuokang Biotechnology Co., Ltd (Jiangsu, China) and allowed to acclimate for 1 week. Female C57BL/6 mice (20 ± 2 g) aged 6–8 weeks were housed individually with free access to food (standard laboratory mouse pellets) and water, in a room with controlled temperature (23°C) and relative humidity (50%), under a 12 h on/12 h off light–dark cycle room lighting. All animal care and use procedures were approved by the Institutional Animal Care and Use Committee of Nanjing University of Science and Technology (IACUC-NJUST-2022-0312), and also followed the guidelines of the National Research Council'Guide for the Care and Use of Laboratory Animals.

The dorsal skin of the mice was shaved, and a 2-day observation period was conducted to confirm the absence of pre-existing skin damage. Mice were randomly assigned to four groups: (1) normal group (Control), neither UV exposure nor any treatment; (2) UVB-irradiated group (UVB), which received UVB exposure alone; (3) Low dose of EPS group (UVB + LP), which received 20 mg/mL EPS before UVB exposure; (5) High dose of EPS group (UVB + HP), which received 40 mg/mL EPS before UVB exposure. The EPS solution was topically applied on the skin of mice at doses of 4 or 8 mg/mouse (in 0.2 mL PBS; 15 min prior to each UVB exposure). For UVB irradiation of back skin, mice were anaesthetized by i.p. Injection of a mix of ketamine (0.08 mg/g body weight) and xylazine (0.008 mg/g body weight) (Navarro et al., 2021). Mice were positioned 10 cm below the UVB light source and irradiated for 20 min, resulting in a dose of 240 mJ/cm^2^. The shaved area on the back of the mice, about 2 cm × 3 cm, was exposed to UVB irradiation, while the other parts of the mice were covered with aluminum foil. The eyes were then covered with vitamin A eye cream to prevent dryness. The UVB irradiation was given three times. After the final irradiation, all mice were euthanized by carbon dioxide inhalation.

### 2.14 Skin injury assessment

Skin manifestations of erythema, edema, roughness and skin wrinkle were respectively scored as 0 (None), 1 (Mild), 2 (Moderate), and 3 (Severe) to evaluate damage level of UVB-challenged skin. Details are shown in Supplementary Table S1 which has been modified based on the previous study (Chen Y. et al., 2022). The histological changes were observed under an optical microscope (DS-Ri2, NIKON, Japan). H&E images were semi-quantitatively analyzed using Image Pro Plus 5.0 (Media Cybernetics, Rockville, MD, United States) to calculate the epidermal thickness per mouse. Collagen content was assessed by measuring the percentage of blue-stained area across Masson’s trichrome-stained sections.

### 2.15 Quantitative real-time PCR

Total RNA was extracted using Trizol and cDNA was synthesized. QRT-PCR was performed with SYBR Green real time PCR mix. The reaction was then conducted using a Real-Time PCR System (7,300 Plus, Applied Biosystems, United States). Gene expression was quantified using the ΔΔCT method. The primers used in this study are listed in Supplementary Table S2.

### 2.16 NMR-based metabolomic profiling

Metabolites were extracted in Control (Control group), UVB (UVB group), and EPS (40 mg/mL of EPS-pretreated group) following previously reported protocols (Liu et al., 2022). Briefly, approximately 200 mg of skin tissues were homogenized and then mixed with 0.6 mL of ice-cold extraction solution. Subsequently, the mixtures were centrifuged at 12,000 g for 10 min. Acetonitrile in the supernatant was evaporated and the remaining residues were freeze-dried. For NMR analysis, the lyophilized samples were dissolved in 600 μL of D_2_O and subjected to a ^1^H NMR experiment using a 500 MHz NMR spectrometer (Bruker, Karlsruhe, Germany). The basic parameters were as follows: relaxation delay: 4 s; spectral width: 20 ppm; number of scans: 32; temperature: 298 K; acquisition time:3.28 s; PULPROG: cpmgpr1d. Metabolites were assigned by Chenomx NMR suite 8.1 (Chenomx Inc., Edmonton, Canada), assisted by querying public metabolomics databases such as Human Metaolome Database (HMDB, http://www.hmdb.ca) and Madison-Qingdao Metabolomics Consortium Database (MMCD, http://mmcd.nmrfam.wisc.edu).

### 2.17 Acute toxicity study of the EPS

The safety profile of the EPS was evaluated based on a previous study with modifications (Anna de Breij et al., 2018). Female C57BL/6 mice aged 6–8 weeks were used in the study. For the acute toxicity study, the EPS was applied uniformly over the shaved skin of the back of the mice in a single dose of 200 mg/kg or 400 mg/kg. After 24 h, the remaining EPS was washed away. After 14 days, animals were euthanized using CO_2_. Tissues of liver, lung, kidney and heart of mice per group for histopathologic studies. Serum obtained from blood samples was used to estimate aspartate transaminase (AST), alanine transaminase (ALT), creatine phosphokinase (CK) and lactate dehydrogenase (LDH) for all the groups, respectively.

### 2.18 Statistical analysis

Data were presented as means ± SD. Statistical analysis was performed by Student’s *t* test or one-way analysis of variance (ANOVA). All calculations were performed using GraphPad Prism 7.0 (GraphPad Software, San Diego, CA). Statistical significance was set at the 0.05 level.

## 3 Results

### 3.1 Identification of a bacterial strain and the EPS production

**FIGURE 1:**
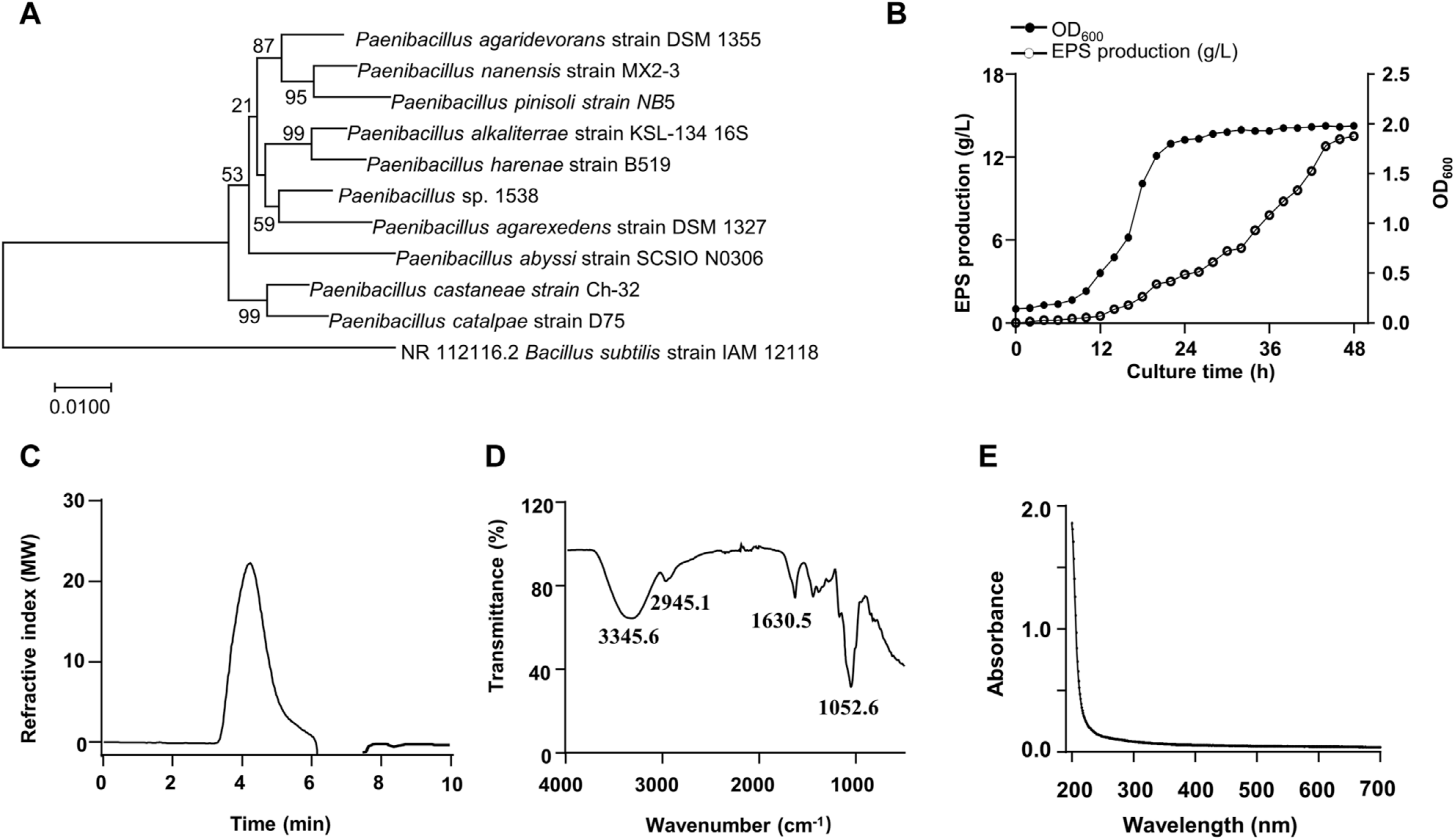
Phylogenetic tree of the isolated strain 1538 and Physiochemical characterization of the EPS. **(A)** Phylogenetic analysis. **(B)** Growth curve and the EPS production by *Paenibacillus* sp.1538. **(C)** GPC chromatogram of the EPS. **(D)** FT-IR spectrum of the EPS. **(E)** UV-vis spectrum of the EPS.

A bacterial strain 1538 producing a large number of EPS was isolated from a forest soil sample. The strain had slightly translucent colonies with thick mucoid. The 16s rDNA sequence was compared with other bacteria through BLAST. The results suggested that the closest species with 99.9% similarity was *Paenibacillus agarexedens*, and a phylogenetic tree was constructed by the neighbor-joining algorithm (Figure 1A). Then, the isolate was designated as *Paenibacillus* sp. 1538. Growth curve (OD_600_) was measured in real-time. The exponential growth phase started after 8 h of incubation, and the stationary phase initiated at 24 h (Figure 1B). The EPS biosynthesis started at the middle of the exponential growth phase, and the optimal EPS production of 13.5 g/L was achieved after 46 h at pH 8.0°C and 30°C. The total sugar content of purified EPS was estimated to be 95.7% (w/w), total protein was 0.1% (w/w), and traces of inorganic salts. Analyzed by GPC, the Mw of the EPS is about 5.48 × 10^3^ kDa (Figure 1C), according to the standard curve (Supplementary Figure S1). The FT-IR spectrum of the EPS exhibited characteristic absorption bands of glycans (Figure 1D). The broad absorption band ranging from 3,500 to 3,000 cm^−1^ arises due to O-H stretching vibrations indicating inter- and intra-molecular hydrogen bonds. The band at 2,945.1 cm^−1^ indicated vibrational stretching of C_sp3_–H (Butt et al., 2024). The absorption peak about 1,630.5 cm^-1^ was attributed to O-H bending vibrations (Gong et al., 2024). The UV-vis spectrum of the EPS is illustrated in Figure 1E and no peaks in the range of 260–280 nm were observed, indicating the absence of nucleic acid or proteins.

### 3.2 Structural characteristics of the EPS

#### 3.2.1 Monosaccharide composition and glycosidic linkages of the EPS

The monosaccharide composition of the EPS was identified by High-performance liquid chromatography. The EPS was composed of D-glucose (D-Glc), D-mannose (D-Man), and D-galactose (D-Gal) with the molar ratio of 2:2:1 (Supplementary Figure S2). Methylation analysis was carried out to determine the glycosyl linkage pattern. As shown in Supplementary Figure S3 and Table 1, the GC–MS results indicated that there were five partially methylated alditol acetates (PMAAs) identified as 2,3,4,6-Me_4_ -Gal*p*, 3,4,6-Me_3_-Glc*p*, 2,4,6-Me_3_-Glc*p*, 2,4,6-Me_3_-Man*p*, and 4,6-Me_2_-Man*p* in a molar ratio of nearly 1:1:1:1:1.

**TABLE 1:** Methylation analysis of the EPS.

PMAAs^a^	Type of linkages	Mass fragments (m/z)
2,3,4,6-Me_4_-Gal*p*	Gal*p*-(1→	87, 101, 117, 129, 145, 161, 205
3,4,6-Me_3_-Glc*p*	→2)-Glc*p*-(1→	87, 129, 161, 189
2,4,6-Me_3_-Glc*p*	→3)-Glc*p*-(1→	87, 101, 117, 129, 161, 233
2,4,6-Me_3_-Man*p*	→3)-Man*p*-(1→	87, 101, 117, 129, 161, 233
4,6-Me_2_-Man*p*	→2,3)-Man*p*-(1→	87, 101, 129, 161, 201, 261

^a^ Partially methylated alditol acetates.

#### 3.2.2 NMR analysis of the EPS

**FIGURE 2:**
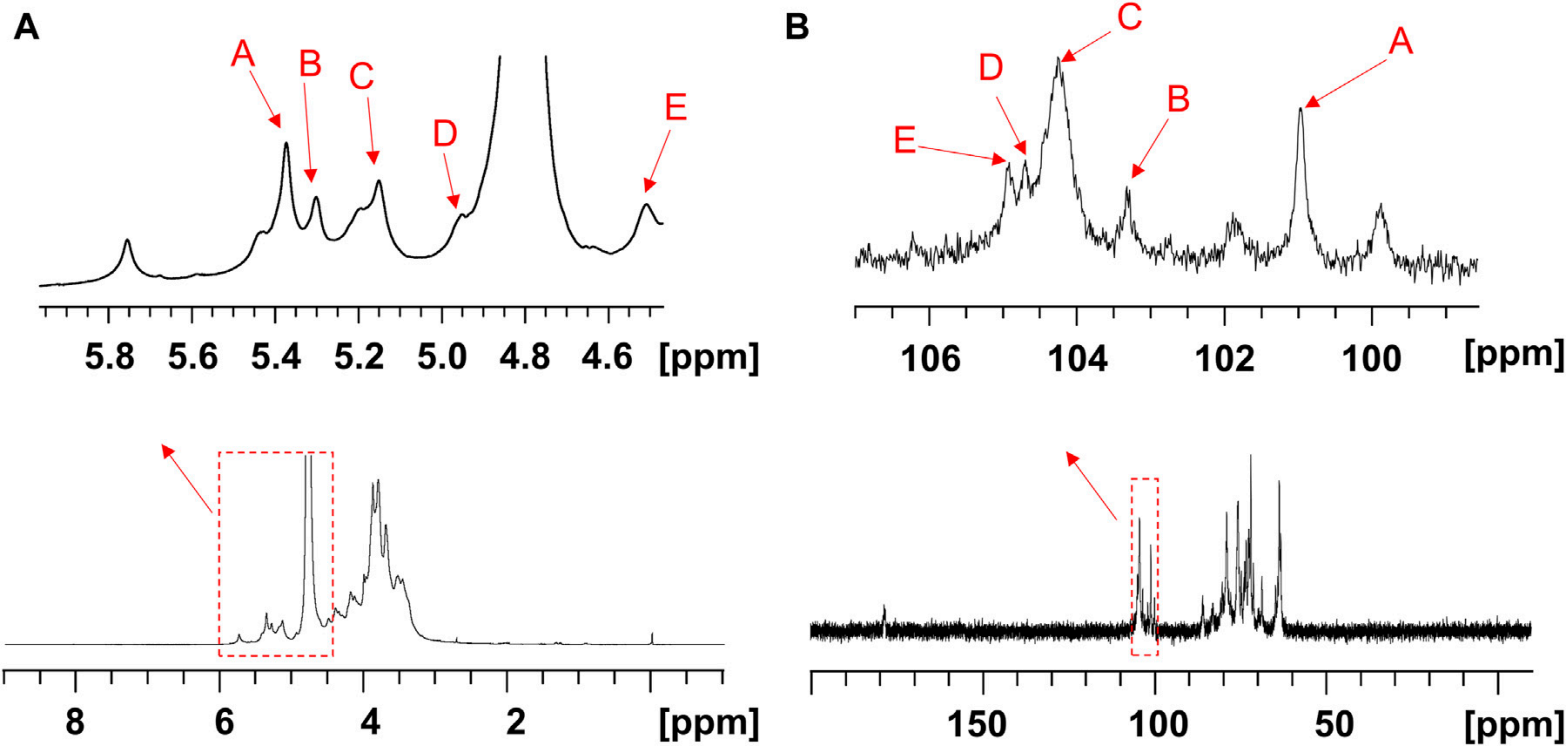
1D NMR spectra of the EPS (500 MHz, D_2_O). The **(A)** ^1^H, **(B)** ^13^C NMR spectra of the EPS.

**FIGURE 3:**
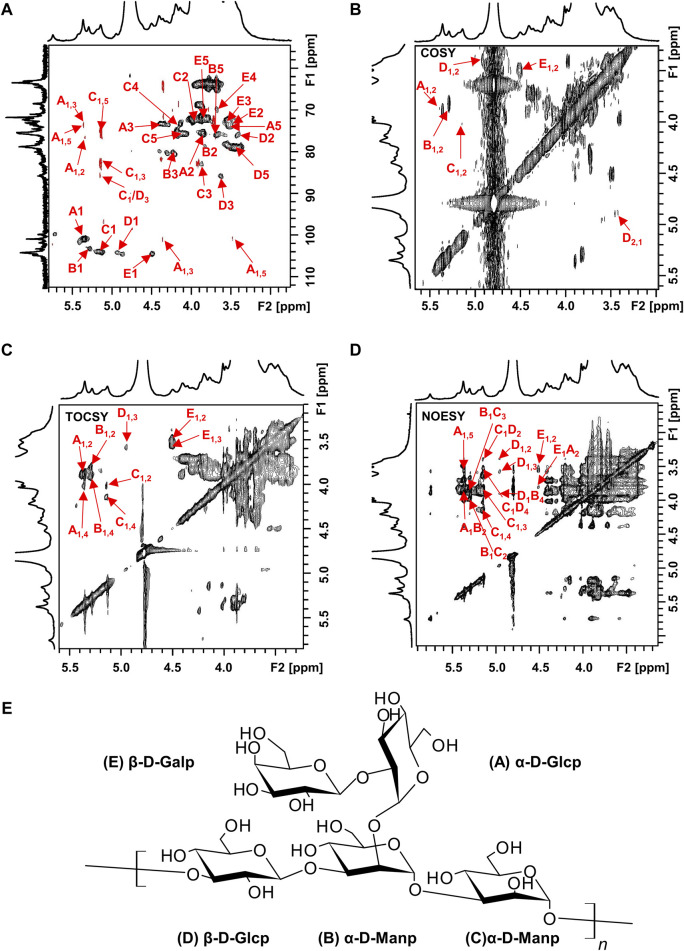
2D NMR spectra of the EPS (500 MHz, D_2_O). **(A)** HSQC (black) and HMBC (red), **(B)** COSY, **(C)** TOCSY, and **(D)** NOESY NMR spectrum of the EPS. **(E)** Chair conformation of the EPS.

The structure of the EPS was established based on 1D, 2D heteronuclear NMR spectra and homonuclear NMR spectra. The ^1^H NMR spectrum (Figure 2A) recorded at 500 MHz showed signals in the anomeric region at 5.37, 5.29, 5.15, 4.95 and 4.50 ppm, designated as A, B, C, D, and E, respectively. Three of the signals between 5.15 and 5.37 ppm were assigned to α-glycosidic bonds, and another two signals located in 4.50 ppm and 4.95 ppm region belonged to a β-glycosidic linkage (Zhang et al., 2019; Sun et al., 2023). The minor anomeric signals at 5.43 ppm and 5.75 ppm were attributed to a non-specifically characterized polysaccharide chain breakage, which were almost completely absent in the ^1^H NMR spectrum of a milder hydrolyzed EPS sample (Supplementary Figure S4). The ^13^C NMR spectrum (Figure 2B) recorded showed five anomeric signals at 101.0, 103.3, 104.4, 104.7, and 104.9 ppm. Coherence and multi-bond correlation between C and H were detected via HSQC and HMBC (Figure 3A). On the basis of the anomeric proton assignments, the anomeric carbon signals were assigned from the HMQC spectrum. The signals at 101.0, 103.3, 104.4, 104.7 and 104.9 ppm corresponded to the anomeric carbons of A, B, C, D and E sugar units respectively. To find the correlations between protons, we performed COSY, TOCSY and NOESY analyses (Figures 3B–D). The chemical shifts of H-2 can be obtained by ^1^H-^1^H COSY spectrum based on the correlation between H-1 and H-2. The complete signals of all hydrogens on one sugar ring can be assigned through sequential analysis by analysis of COSY and TOCSY. Starting from anomeric hydrogens, H-1 of Gal (E) is located at 4.50 ppm. The cross peak at 4.50/3.48 ppm indicates that the chemical shift of H-2 is 3.48 ppm. The glycosyl sequence was easy to be analyzed via HMBC and NOESY spectra. Similarly, the chemical shift of H-3 was determined to be 3.59 ppm. Combined with the cross peak between the (E) H-1 and (A) H-2 at 4.50/ 3.87 ppm, the side chain of the EPS was determined to be composed of (E) β-D-Gal*p* (1→2) -(A) α-D-Glc*p* (1→2 and the branch point is (B)Man. Based on the correlations NOESY spectrum (Figure 3D), it could be deduced that the backbone of the EPS composed of →3) -(D) β-D-Glc*p* (1→3) -(B) α-D-Man*p* (1→3) -(C) α-D-Man*p* (1→. Then these hexapyranose spin systems were distinguished according to cross-peaks in 2D NMR and accurately assigned chemical shifts of each glycoside. Correlations were marked in these plots on the basis of all available information (Table 2). The chair conformation of the EPS was shown in Figure 3E. The NMR signals were assigned referring to online tools and databases (Kapaev and Toukach, 2015; Toukach and Egorova, 2016; Klukowski and Schubert, 2019). Combining all results from the monosaccharide composition, the methylation analysis, and the 1D and 2D NMR spectroscopy, the structure of linked pattern was proposed for the EPS was as follows: →3)-β-D-Glc*p* (1→3) (β-D-Gal*p* [1→2]-α-D-Glc*p* [1→2])-α-D-Man*p* (1→3)-α-D-Man*p* (1→.

**TABLE 2:** Peak assignments of ^1^H and ^13^C NMR spectra of the EPS (ppm).

	Chemical shifts (ppm)					
	H1/C1	H2/C2	H3/C3	H4/C4	H5/C5	H6/C6
[A] →2) α-D-Glc*p* (1→	5.37	3.87	4.34	4.02	3.47	3.75
	101.0	76.1	73.4	72.2	73.7	63.9
[B] →2,3) α-D-Man*p* (1→	5.29	3.80	4.20	3.90	3.67	—
	103.3	78.5	80.4	71.6	75.9	63.8
[C] →3) α-D-Man*p* (1→	5.15	4.01	3.90	4.14	4.07	—
	104.4	71.9	82.4	73.1	75.6	63.8
[D] →3) β-D-Glc*p* (1→	4.95	3.40	3.62	3.49	3.41	—
	104.5	75.6	85.9	73.6	78.7	63.6
[E] β-D-Gal*p* (1→	4.50	3.48	3.55	3.67	3.83	3.99
	104.7	72.4	73.2	70.1	72.5	63.6

### 3.3 Effects of the EPS and UVB on cell viability

**FIGURE 4:**
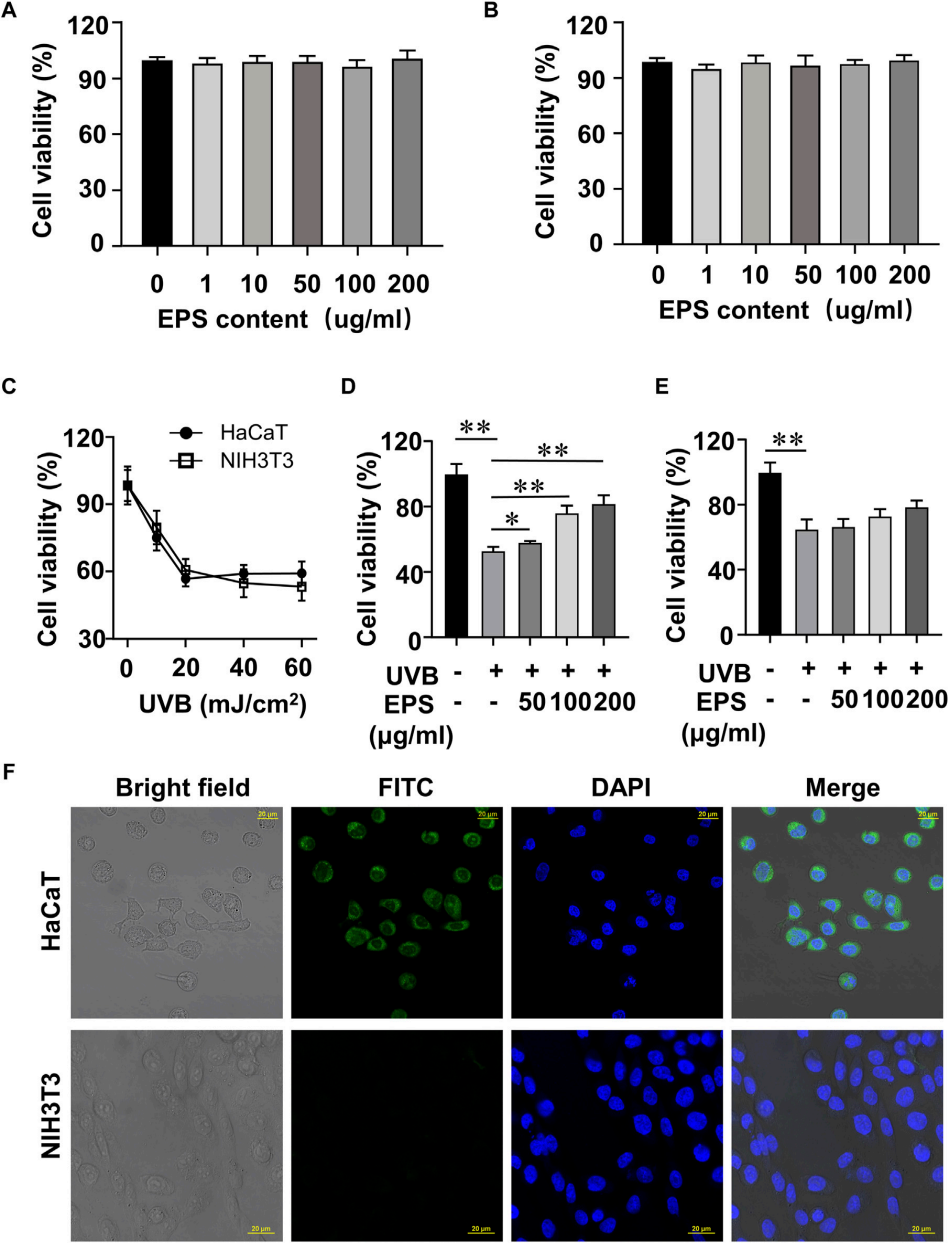
Protective effect of the EPS on UVB-induced cytotoxicity in HaCaT cells and NIH3T3 cells. Cytotoxicity assay of the EPS in **(A)** HaCaT cells and **(B)** NIH3T3 cells. **(C)** Cell viability of HaCaT cells and NIH3T3 cells at different UVB exposure dose (0–60 mJ/cm^2^). Cell viability of HaCaT cells **(D)** and NIH3T3 cells **(E)** with different EPS concentration (0–200 μg/mL). **(F)** Representative fluorescence microscopy images showing HaCaT cells and NIH3T3 cells treated with FITC-labeled EPS (100 μg/mL). Scale bar = 20 µm. Data were presented as mean ± SD, with N = 3. Statistical analysis was performed by one-way ANOVA. *p < 0.05, **p < 0.01.

In order to screen the biological activities of EPS, we conducted experiments to examine the viability of UVB-exposed cultured cells. At doses ranging from 1 to 200 μg/mL EPS had no harmful effects on HaCaT cells and NIH3T3 cells (Figures 4A, B). As shown in Figure 4C, the cell viability of HaCaT cells and NIH3T3 cells reached 53.15% and 60.87% at a dose of 20 mJ/cm^2^ of UVB exposure, respectively. Thus, the photoprotective efficacy of the EPS on UVB-induced cells was assessed with a dose of 20 mJ/cm^2^. Pre-administration of the EPS reduced UVB-induced cell death in HaCaT cells (Figure 4D), but not in NIH3T3 cells (Figure 4E), indicating a possibility that the EPS may specifically target some cells in the skin to protect against UV damage. Via confocal microscopy analysis, it showed that the EPS with fluorescence were mainly localized in the membranes in HaCaT cells, but not in NIH3T3 cells (Figure 4F). These results suggest that keratinocytes may serve as the primary target for the EPS in protecting the skin from UVB-induced photodamage.

### 3.4 Effect of the EPS on UVB-induced ROS production and oxidative stress in HaCaT cells

**FIGURE 5:**
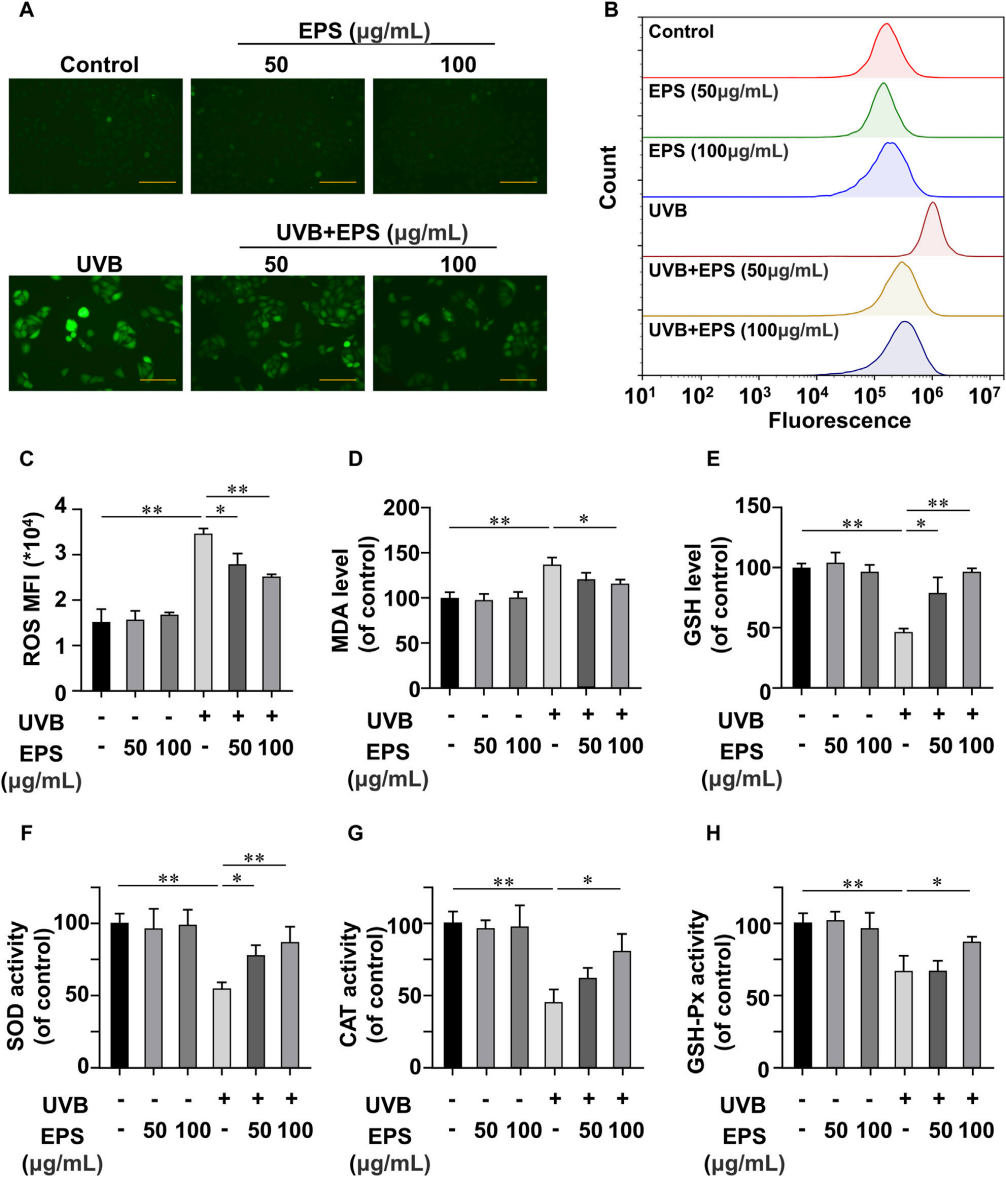
Antioxidant Effects of the EPS on HaCaT cells **(A)** Representative photomicrographs of fluorescent cells are shown. Scale bar = 100 μm. **(B)** Intracellular ROS level detected by flow cytometry. **(C)** ROS levels in each group (MFI: mean fluorescence intensity). Effect of different EPS doses on **(D)** GSH, **(E)** MDA levels and **(F)** SOD, **(G)** MDA, and **(H)** GSH-Px activities in UVB-irradiated HaCaT cells. Data were presented as mean ± SD, with N = 3. Statistical analysis was performed by one-way ANOVA. *p < 0.05, **p < 0.01.

The oxidative stress induced by the undesirable intracellular accumulation of ROS is a major cause of cell toxicity, reducing ROS accumulation presents a potential approach for safeguarding the skin against photodamage (Carrasco et al., 2015; Guan et al., 2021; Li et al., 2022b; Shetty et al., 2023). We then investigated the UVB-induced changes of endogenous ROS using fluorescence microscopy and flow cytometry. UVB irradiation stimulated cellular ROS generation in HaCaT cells, and the EPS markedly decreased ROS levels (Figures 5A, B) in a dose dependent manner (Figure 5C). ROS overproduction can lead to lipid peroxidation, and MDA is a biomarker of lipid peroxidation (Han et al., 2023). As shown in Figure 5D, UVB caused an increase of MDA content, which was inhibited by the EPS. We also observed that the EPS could influence the intracellular antioxidant system by increasing the levels of endogenous antioxidant GSH (Figure 5E). While the activities of SOD, CAT, and GSH-Px were substantially reduced in the UVB group, the EPS significantly recovered their activities (Figures 5F–H). These results suggested that the EPS could reduce UVB-induced oxidative stress in HaCaT cells.

### 3.5 Effects of the EPS on skin morphology and histochemical damage in UVB-exposed mice

**FIGURE 6:**
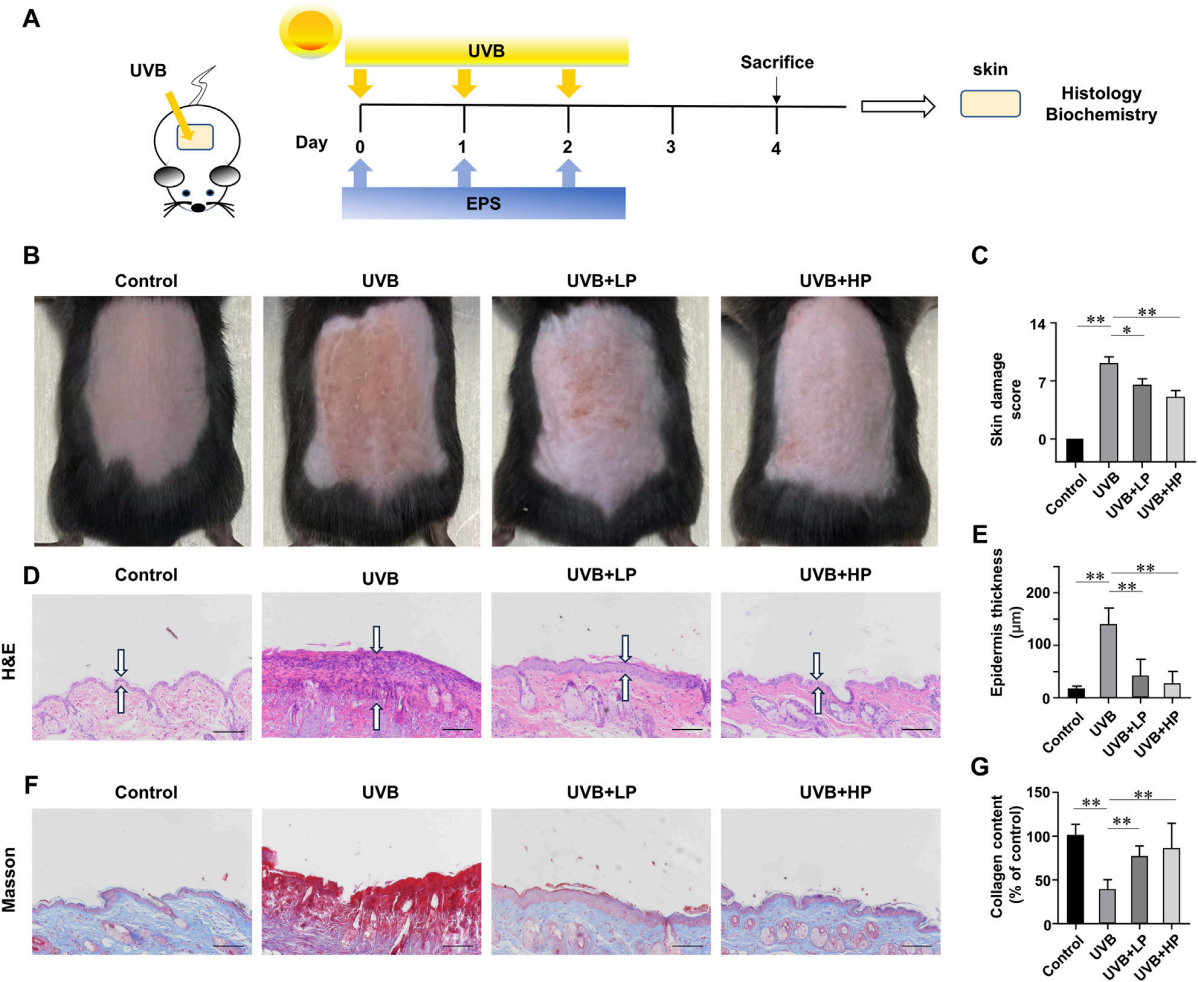
Protective effects of EPS on UVB-induced skin injury. **(A)** Design of the UVB Irradiation study. **(B)** Macroscopic evaluation of dorsal skin. **(C)** Skin damage scores were also calculated statistically. **(D)** H&E staining of skin. Double arrows indicate the epidermis thickness. **(E)** Quantification of epidermal thickness from H&E-stained sections from the indicated groups. **(F)** Masson staining of skin. **(G)** The collagen fraction was quantified from the Masson trichrome-stained skin sections (n = 5). Scale bar = 100 µm. Data were presented as mean ± SD. Statistical analysis was performed by one-way ANOVA. *p < 0.05, **p < 0.01.

We then investigated whether the EPS possessed an anti-UVB effect on the skin of mice. The schedule of UVB exposure was given in Figure 6A. The macroscopic effects on UVB-irradiated mice dorsal skin and damage score were shown in Figures 6B, C. The UVB group appeared typical photoaging characteristics such as erythema, edema, roughness and significant wrinkles. While a low dose of EPS topical application alleviated the deleterious effects of UVB irradiation slightly, the skin appearance of mice in the high-dose treatment of the EPS group was markedly improved, showing no signs of dark-brown color or sunburns, which was almost close to the Control group. Hematoxylin and eosin staining results were exhibited in Figure 6D. In the EPS groups, epidermal thickness was maintained close to basal levels, indicating that skin health was improved by the EPS topical application (Figure 6E). To examine changes in collagen fibers, dorsal skins were stained with Masson’s trichrome stain. As shown in Figure 6F, collagen fibers stained with blue dye were abundant in the Control group. After UVB irradiation, collagen fibers in the dorsal dermis were significantly decreased (Figure 6G). The EPS-treated groups recovered the degradation of collagen fibers in a dose-dependent manner. The results suggested that the EPS relieved erythema, skin thickening and collagen loss due to UVB exposure.

### 3.6 Anti-inflammatory and antioxidant effects of the EPS on UVB-exposed mice

**FIGURE 7:**
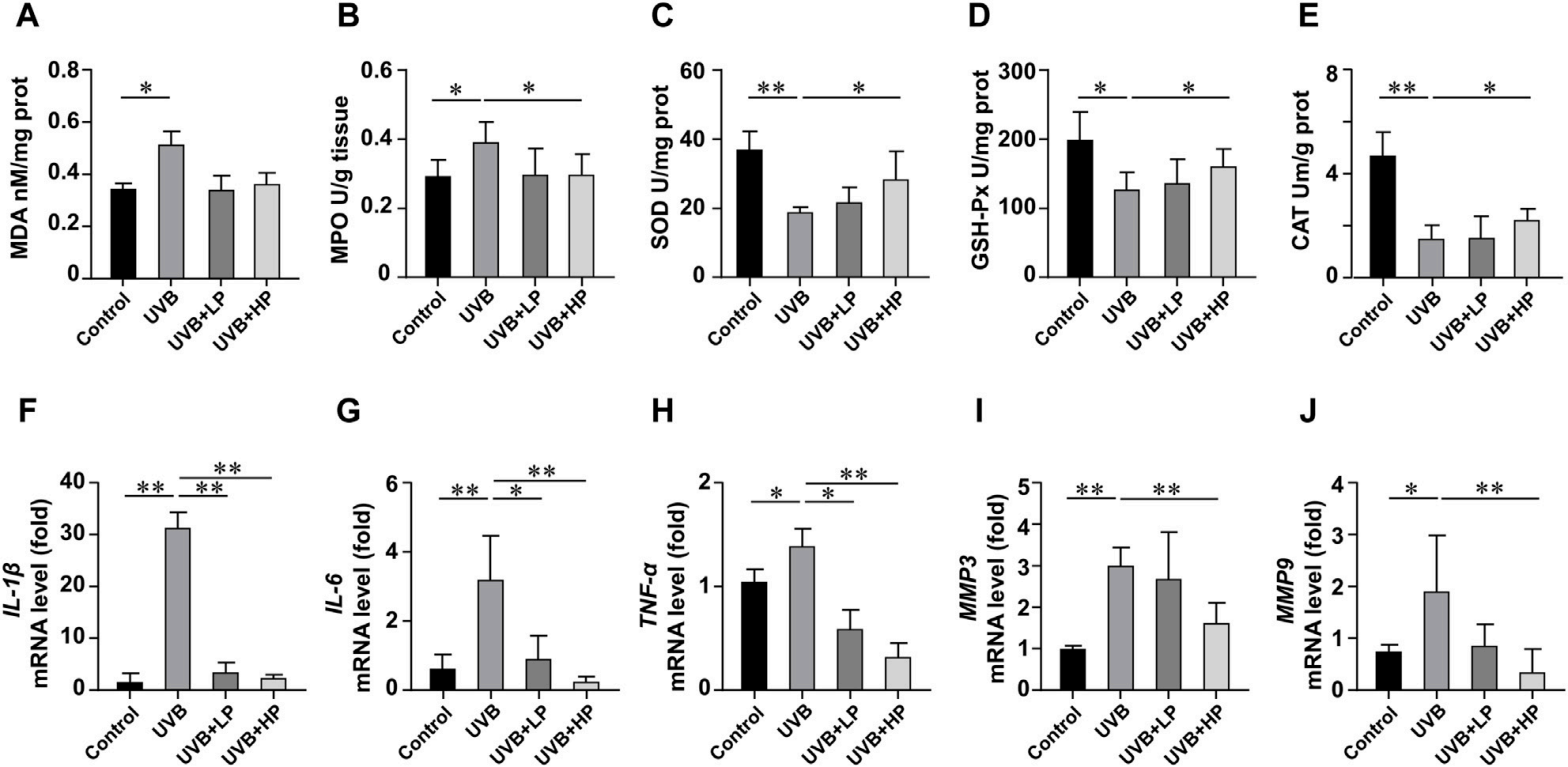
Analysis of antioxidant activities and gene expression in skin tissues. Content of **(A)** MDA, and activities of **(B)** MPO, **(C)** SOD, **(D)** GSH-Px and **(E)** CAT in skin tissues. Gene expression of **(F)** *IL-1*β, **(G)** *IL-6,* **(H)** *TNF-α*, **(I)** *MMP3* and **(J)** *MMP9* in skin tissues. Data were presented as mean ± SD, with N = 6. Statistical analysis was performed by one-way ANOVA. *p < 0.05, **p < 0.01.

In Figures 7A–E, UVB radiation notably increased MDA and MPO levels, while decreasing SOD, GSH-Px and CAT activities compared to the Control group. These biochemical markers related to oxidative stress showed an improvement with the EPS treatment. In addition, the EPS reduced oxidative stress-induced skin inflammation. In Figures 7F–H, data determined by qRT-PCR revealed a significant increase in *IL-1β*, *IL-6*, and *TNF-α* mRNA expression in the UVB group compared to the Control group. The EPS treatment decreased *IL-1β*, *IL-6*, and *TNF-α* mRNA expression induced by UVB irradiation in a dose-dependent manner. The EPS reduced the expression of *MMP3* and *MMP9* (Figures 7I, J), leading to a decrease in visible signs of photodamage such as wrinkles and loss of elasticity in the skin.

### 3.7 Metabolic analysis for the EPS-treat mice under UVB-exposure

**FIGURE 8:**
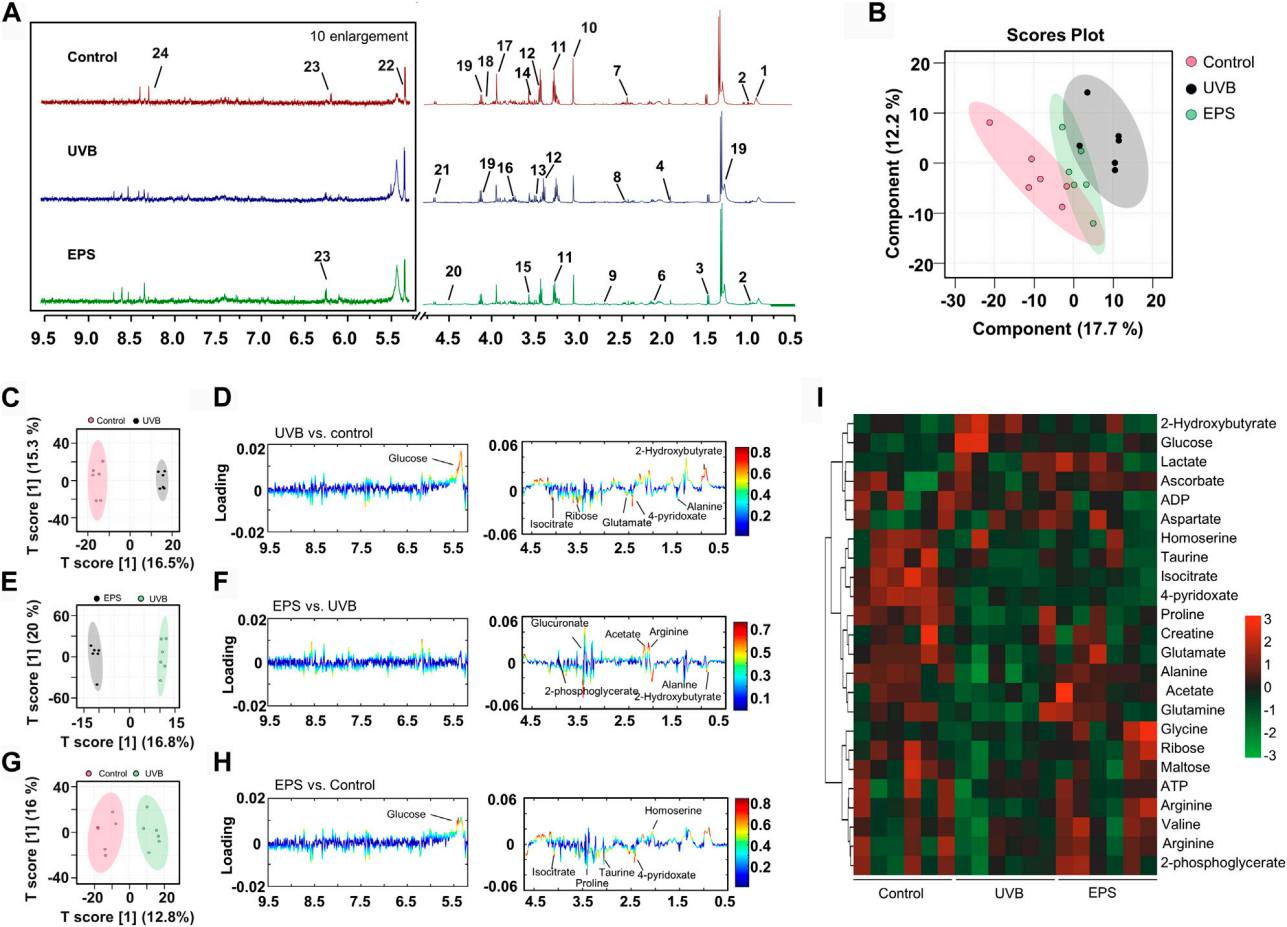
Metabolomics analysis of the skin of mice. **(A)** Typical ^1^H NMR spectrum of skin of extracts with assigned metabolites from mice, 500 MHz, D_2_O. **(B)** Score plot for PLS-DA analysis. **(C, D)** Score plots and color-coded coefficient loadings plots corresponding OPLS-DA analysis of Control group and UVB group. **(E, F)** Score plots and color-coded coefficient loadings plots corresponding OPLS-DA analysis of Control group and the EPS group. **(G, H)** Score plots and color-coded coefficient loadings plots corresponding OPLS-DA analysis of the UVB group and the EPS group. **(I)** Heat map of 24 metabolites from the Control group, UVB group, and the EPS group. n = 6.

To confirm the effects of the EPS on protecting UVB injury, we performed a metabolomics analysis across three groups (Control, UVB and EPS groups). Typical ^1^H NMR spectra with assigned metabolites of skin extracts were presented in Figure 8A. In the principal Partial least squares Discriminant Analysis (PLS-DA) score plot, Control group and UVB group were both well separated from control group, whereas the Control group overlapped with the EPS group (Figure 8B), suggesting similar metabolic changes in both groups. In the OPLS-DA score plot and color-coded loading plots, the UVB group showed significant shifts of the metabolites (either increase or decrease) compared to Control group (Figures 8C, D). However, the EPS treatment reversed the metabolite alterations caused by UVB partially (Figures 8E, F). The metabolite alterations were less pronounced between the EPS group and the Control group (Figures 8G, H). A heatmap based on the relative abundances of the metabolites was also generated to better display the separation in metabolites between the UVB group and the Control group, as well as the similarity in metabolites among the EPS-pretreated group and Control group (Figure 8I). The statistical analysis of major metabolites was performed and shown in Table 3. In comparison to the control group, the UVB-exposed mice demonstrated a significant decrease in alanine, 4-pyridoxate, homoserine, proline, ribose, isocitrate, and maltose levels within their skin tissues, while an increase in 2-Hydroxybutyrate and glucose levels. The significant elevations of the glucose in the skin tissues of UVB-exposed mice, serving as one of the signs of skin aging (Danby, 2010), which could be ameliorated by the EPS. The other significantly changed levels of metabolites in the UVB group, such as 2-Hydroxybutyrate, alanine and glucose were partially reversed after the EPS pretreatment, suggesting good protection of the EPS against UVB-induced metabolites disorder in skin. These results indicated that the EPS reverses the shifts of metabolic profiles of the skin in UVB-exposed mice.

**TABLE 3:** Identified metabolites in skin tissues and their fold-change values and associated *p* values.

NO	Metabolites	UVB/Ctrl		EPS/UVB		EPS/Ctrl	
		Log (FC)^a^	*p* ^b^	Log (FC)	*p*	Log (FC)	*p*
1	2-Hydroxybutyrate	0.17	**	−0.13	*	0.04	
2	Valine	−0.06		0.09		0.04	
3	Alanine	−0.13	**	0.11	**	−0.02	
4	Arginine	−0.11		0.14		0.03	
5	Acetate	−0.1		0.16	*	0.06	
6	Homoserine	−0.23	*	−0.01		−0.24	*
7	4-pyridoxate	−0.38	**	−0.04		−0.41	**
8	Glutamate	−0.12		0.09		−0.05	
9	Aspartate	0.06		−0.01		0.05	
10	Creatine	−0.04		−0.02		−0.05	
11	Taurine	−0.25		0.01		−0.24	
12	Proline	−0.12	**	0.03		−0.11	*
13	Glucuronate	−0.13		0.14	*	0.01	
14	Glycine	−0.02		0.18	*	0.16	*
15	Ribose	−0.11	*	0.09		−0.01	
16	Glutamine	−0.05		0.06		0.01	
17	2-phosphoglycerate	−0.08		0.11	*	0.02	
18	Isocitrate	−0.24	*	0.02		−0.21	
19	Lactate	0.04		0.03		0.07	
20	Ascorbate	0.04		0.04		0.08	
21	Maltose	−0.12	*	0.04		−0.08	
22	Glucose	0.2	*	−0.1		0.11	
23	ADP	−0.14		0.04		−0.13	
24	ATP	−0.07		0.18		−0.1	

^a^ Red/blue colors denote increased/decreased levels of characteristic metabolites.

^b^ *p*-values were calculated based on a parametric Student’s t-test or a nonparametric Mann–Whitney test (dependent on the conformity to normal distribution). *p < 0.05, **p < 0.01.

### 3.8 Acute toxicity of the EPS

The EPS had no potential adverse effects associated with systemic toxicity after directly administered to the skin. Specifically, we observed no significant dose-dependent histopathologic changes or apoptosis in the liver, lung, kidney, and heart tissues of all treatment groups (Supplementary Figure S5A, B). None of the biochemical parameters differed significantly in the treatment group from the control group in mice (Supplementary Figure S5C). It may be concluded that the EPS up-to certain limits is safe for animals.

## 4 Discussion

Excessive exposure to sunlight can cause damage to the skin, leading to sunburn, photo-aging and skin cancer (Hao et al., 2019). Most commercial sunscreens incorporate organic or inorganic UV filters to shield against skin damage. Despite their efficacy in preventing cutaneous phototoxicity, concerns have emerged regarding cellular exposure to these filters (Deng et al., 2015). Many studies have reported the potential of EPSs as natural sunscreen agents due to their UV-absorbing and antioxidant properties (Lee et al., 2021). Additionally, these polysaccharides have also been shown to prevent skin aging and improve skin hydration (Chen et al., 2016; Li et al., 2022a). Based on these properties, the addition of natural ingredients to skin-care cosmetics has been a predictable trend.

UVB irradiation significantly decreased cell viability in both fibroblasts and keratinocytes. The EPS reduced UVB-induced cell death in HaCaT cells, but not in NIH3T3 cells, suggesting that keratinocytes may be the primary target for the protective effects of the EPS against UVB-induced photodamage on the skin, which was confirmed by a specific binding of the EPS to HaCaT cells. HaCaT cells constitutively express mRNA of membrane-bound TLR1, 2, 4, Dectin-1 and DC-SIGN, which can recognize polysaccharide component. Curdlan 1,3-β-glucan enhances wound healing by increasing human keratinocyte migration and proliferation in a dectin-1-dependent manner (Gao et al., 2021). Conversely, several studies have shown that NIH3T3 cells shows no endogenous activities of TLR1,2,4, DC-SIGN and Dectin-1. This difference in receptor expression could possibly support a highly specific bonding to the EPS (Taylor et al., 2004; Burger-Kentischer et al., 2010; Huang et al., 2013; Zhao et al., 2013). UVB overexposure leads to increased intracellular ROS levels and oxidative stress, which are the main causes of UVB-induced damage (Carrasco et al., 2015). ROS is presumably the most frequent intrinsic source of DNA damage. Mildly damaged cells may repair the DNA lesions and recover. However, upon excessive ROS-induced massive DNA damage, cells fail to undergo productive DNA repair and trigger the cell death response (Matt and Hofmann, 2016). Oxidative stress disrupts the prooxidant-antioxidant balance in the body, leading to disturbances in redox signaling. Enzymatic (SOD, CAT, GSH-Px) and non-enzymatic (GSH) antioxidant molecules play crucial roles in protecting cells from oxidative damage. Pretreatment with the EPS increased HaCaT cell viability, decreased the intracellular levels of ROS, enhanced the activities of enzymatic antioxidants (SOD, CAT and GSH-Px) and GSH levels, and suppressed the release of MDA. These findings suggest that the EPS could mitigate UVB-induced cell damage by enhancing the antioxidant defenses of HaCaT cells.

Erythema, epidermal thickness and collagen are usually key parameters in evaluating UVB-related skin damage. Macroscopically, application of the EPS topically significantly reduced the skin damage in mice. The EPS reduced epidermal thickening and collagen loss, suggesting a lower degree of overall skin injury. Then, inflammatory cytokines such as TNF-α, IL-6 and IL-1β are involved in immune response and cause photodamage (Li et al., 2021). UVB-induced oxidative stress increases MMPs, including MMP-3 and MMP-9, which degrade collagen, leading to tissue structure damage and promoting wrinkle formation (Kanlayavattanakul et al., 2024). Restoring anti-oxidants is a potential target for drug investigation to suppress UVB-induced tissue injury. The EPS restored antioxidant enzyme activity, indicating that the EPS can counteract the significant increase in UVB-induced oxidative stress, thereby inhibiting collagen degradation.

The metabolic profile results demonstrated that UVB-induced skin damage caused significant metabolic alterations. The significant increase in glucose levels in the skin tissues of UVB-exposed mice leads to the production of advanced glycation end products, contributing to skin aging. Inhibiting advanced glycation may improve skin texture and appearance (Narda et al., 2018). Additionally, studies have shown that the amino acids are not only needed for protein synthesis, but they play specialized roles in maintaining healthy skin (Hou et al., 2020). For example, proline is one of the most abundant amino acids in collagen, and also regulator of collagen synthesis. Alanine plays a general role in water retention in the stratum corneum. Metabolome analysis of skin tissues showed that the EPS effectively regulates the levels of the metabolites associated with photoaging effectors induced by UVB irradiation. This finding provides systematic perspective of protective effect of the EPS on UVB-induced skin damage as well as a basic for further pharmacological and clinical research.

Though we have revealed the EPS as the therapeutic strategy of UVB-induced skin damage, the detailed mechanism is unclear. Beyond the antioxidant effect that has been studied in this study, further exploration will be required to understand detailed mechanism. In addition, further study is necessary to analyze the synergistic effect of the EPS when combined with a synthetic sunscreen. In summary, the EPS exhibited excellent antioxidant properties *in vitro* and *in vivo*, which may provide further insights into the EPS as an attractive therapeutic agent for oxidative stress-related diseases.

## 5 Conclusion

In conclusion, a novel EPS was produced by *Paenibacillus* sp 1538. The EPS was composed of glucose, mannose and galactose residues at a ratio of 2:2:1, with an average molecular weight of 5.48 × 10^3^ kDa. The repeating units of the EPS was identified as: →3)-β-D-Glc*p* (1→3) [β-D-Gal*p* (1→2)-α-D-Glc*p* (1→2)]-α-D-Man*p* (1→3)-α-D-Man*p* (1→. We have demonstrated that the EPS protects against UVB-induced photodamage by inhibiting excessive ROS generation and balancing various antioxidant enzymes, suggesting that the EPS provides a safe and potent option for sunscreen additive.

## Data Availability

The original contributions presented in the study are included in the article/Supplementary Material, further inquiries can be directed to the corresponding author.
