# Supplementary material for: The structural characterization and UV-protective properties of an exopolysaccharide from a Paenibacillus isolate
Source: Front Pharmacol. 2024 Aug 9;15:1434136. doi: 10.3389/fphar.2024.1434136 (PMC11341463; doi:10.3389/fphar.2024.1434136)
Supplement: Supplementary file 1 [file DataSheet1.docx]

***Supplementary Tables:***

**Table S1 Evaluation criteria for acute skin photodamage.**

| **Score** | **Erythema** | **Edema** | **Wrinkles** | **Roughness** |  |
| --- | --- | --- | --- | --- | --- |
| **0** | Similar to the unexposed skin | | | | |
| **1** | Pink | Mild swelling | Slight wrinkles | Mild roughness | |
| **2** | Red | Slightly harden | Moderate wrinkles | Moderate coarseness | |
| **3** | Dark red | Harden | Sever wrinkles | Leather-like coarseness | |

**Table S2 Primer sequence**

| **Gene** | **Forward Primer (5’ to 3’)** | **Reverse Primer (5’ to 3’)** | |
| --- | --- | --- | --- |
| ***GAPDH*** | AGGFMGGTGTGAACGGATTTG | | AGGFMGGTGTGAACGGATTTG |
| ***Tnf-α*** | GGTGCCTATGFMFMAGCCFMTT | | GGTGCCTATGFMFMAGCCFMTT |
| ***IL-1β*** | GCCTTGGGCCTCAAAGGAAAGAATC | | GGAAGACACAGATTCCATGGTGAAG |
| ***IL-6*** | CAGAGGATACCACTCCCAACAGAC | | CTCTGAAGGACTCTGGCTTTGTC |
| ***MMP3*** | GGCCTGGAACAGTCTTGGC | | TGTCCATCGTTCATCATCGTCA |
| ***MMP9*** | CTGGACAGCCAGACACTAAAG | | CTCGCGGCAAGTCTTCAGAG |

***Supplementary Figures:***


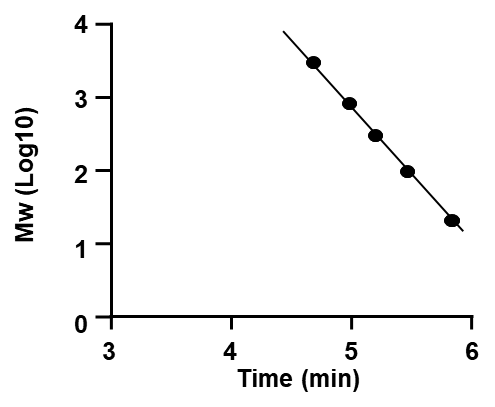


**Fig. S1. Standard curve of Mw.** (y = -1.8778x + 12.262, R² = 0.9997).


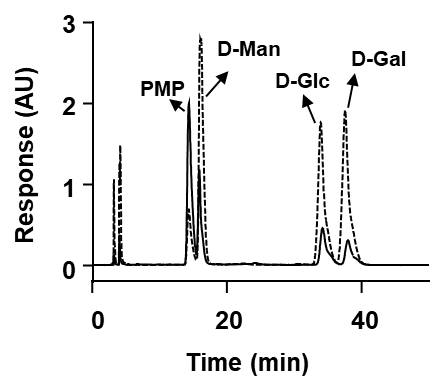


Fig. S2. HPLC chromatograms of the derivatives of component monosaccharides from the EPS and standard monosaccharides.

**
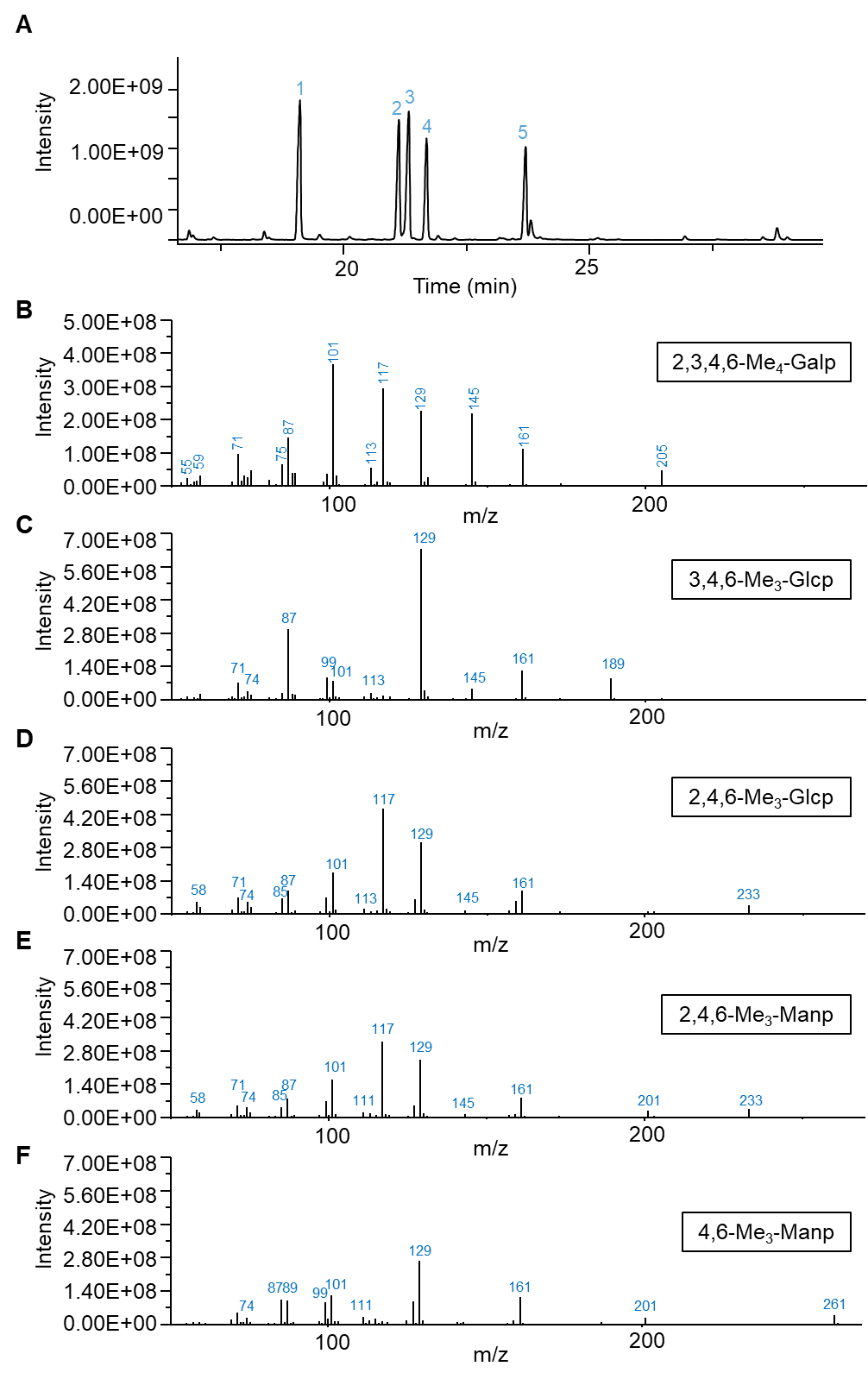
**

**Fig. S3. Methylation analysis**. **(A)** The total ion chromatogram of the EPS in the methylation-GC/MS analysis. All mass fragments of peaks were numbered. (B-F) all the mass spectrometry fragments of PMAAs: **(B)** PMAA-1: 2,3,4,6-Me4-Gal*p*, **(C)** PMAA-2: 3,4,6-Me3-Glc*p*, **(D)** PMAA-3: 2,4,6-Me3-Glc*p*, **(E)** PMAA-4: 2,4,6-Me3-Man*p*, **(F)** PMAA-5: 4,6-Me_2_-Man*p*.


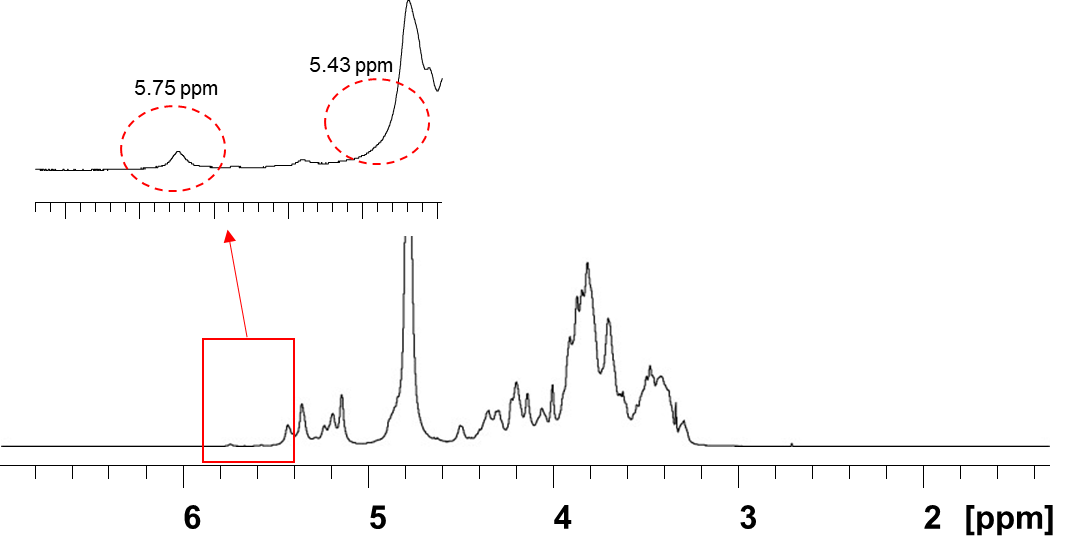


**Fig. S4. The ^1^H NMR spectra of the EPS sample, which was hydrolyzed in 0.5 M TFA at 100 °C for 0.5 h.**


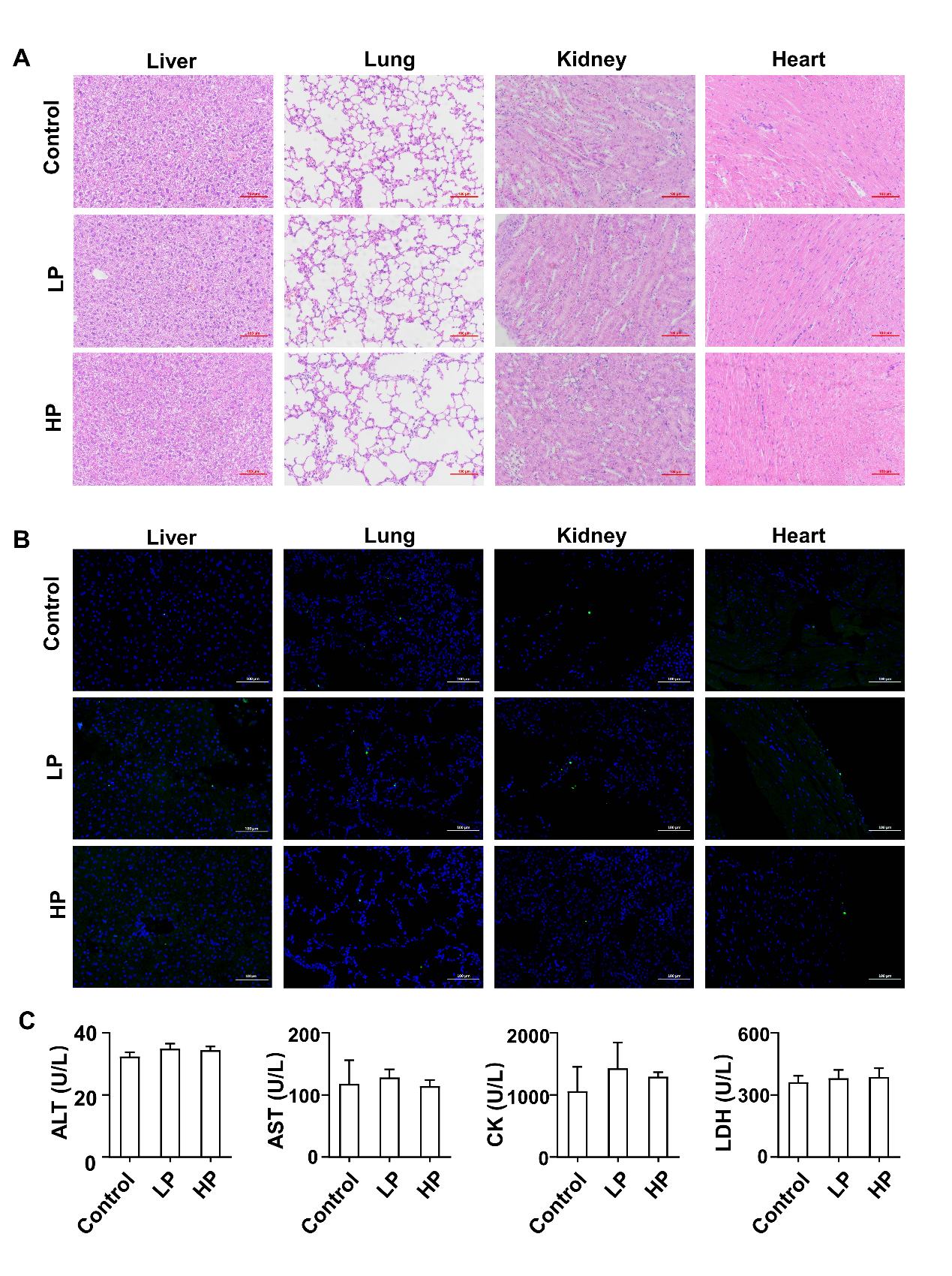


**Fig. S5. Acute toxicity of the EPS. (A)** H&E staining of liver, lung, kidney and heart tissues. Scale bar = 100 µm. **(B)** TUNEL analysis of lung liver, lung, kidney and heart tissue sections. Scale bar = 100 µm. **(C)** Biochemical parameters of ALT, AST, CK and LDH. Data were presented as mean ± SD, with N = 6.
